# Cerebral vs. Cardiovascular Responses to Exercise in Type 2 Diabetic Patients

**DOI:** 10.3389/fphys.2020.583155

**Published:** 2021-01-15

**Authors:** Yu-Sok Kim, Björn J. P. van der Ster, Patrice Brassard, Niels H. Secher, Johannes J. van Lieshout

**Affiliations:** ^1^ Laboratory for Clinical Cardiovascular Physiology, Amsterdam UMC, University of Amsterdam, Amsterdam, Netherlands; ^2^ Department of Medical Biology, Amsterdam UMC, University of Amsterdam, Amsterdam, Netherlands; ^3^ Department of Internal Medicine, Medisch Centrum Leeuwarden, Leeuwarden, Netherlands; ^4^ Department of Anesthesiology, Amsterdam UMC, University of Amsterdam, Amsterdam, Netherlands; ^5^ Department of Kinesiology, Faculty of Medicine, Research Center of the Institut Universitaire de Cardiologie et de Pneumologie de Québec, Université Laval, Québec, QC, Canada; ^6^ Department of Anesthesia, The Copenhagen Muscle Research Center, University of Copenhagen, Copenhagen, Denmark; ^7^ MRC/Arthritis Research UK Centre for Musculoskeletal Ageing Research, School of Life Sciences, University of Nottingham Medical School, Queen’s Medical Centre, Nottingham, United Kingdom; ^8^ Department of Internal Medicine, Amsterdam UMC, University of Amsterdam, Amsterdam, Netherlands

**Keywords:** cardiac output, cerebral blood flow, cerebral oxygenation, cerebral metabolism, diabetes, vascular conductance

## Abstract

The human brain is constantly active and even small limitations to cerebral blood flow (CBF) may be critical for preserving oxygen and substrate supply, e.g., during exercise and hypoxia. Exhaustive exercise evokes a competition for the supply of oxygenated blood between the brain and the working muscles, and inability to increase cardiac output sufficiently during exercise may jeopardize cerebral perfusion of relevance for diabetic patients. The challenge in diabetes care is to optimize metabolic control to slow progression of vascular disease, but likely because of a limited ability to increase cardiac output, these patients perceive aerobic exercise to be more strenuous than healthy subjects and that limits the possibility to apply physical activity as a preventive lifestyle intervention. In this review, we consider the effects of functional activation by exercise on the brain and how it contributes to understanding the control of CBF with the limited exercise tolerance experienced by type 2 diabetic patients. Whether a decline in cerebral oxygenation and thereby reduced neural drive to working muscles plays a role for “central” fatigue during exhaustive exercise is addressed in relation to brain’s attenuated vascular response to exercise in type 2 diabetic subjects.

## Introduction

Animals like the Crucian carp and the aquatic turtle can survive anoxia for extended periods of time (Sick et al., 1982; Lutz et al., 1985; Hochachka and Lutz, 2001; Nilsson and Lutz, 2004), but human brain function depends on continuous delivery of oxygen and nutrients. Thus, interruption of blood supply to the brain for only a few seconds results in loss of consciousness (Rossen et al., 1943; Finnerty et al., 1954; Smith et al., 2011). Accordingly, even minor limitations to cerebral blood flow (CBF) may be critical in preserving oxygen and substrate supply to the brain and in that regard the human brain is challenged by exercise and hypoxia (Kim, 2014). When the brain is activated to perform exercise, the increment in CBF enhances brain oxygenation whereas skeletal muscle oxygenation decreases progressively with work rate. Thus, functional activation of the brain initially leads to hyperperfusion, while the large increase in skeletal muscle blood flow during exercise may be taken to be insufficient (Quistorff et al., 2008).

Reduced exercise tolerance in type 2 diabetes mellitus (T2DM) is incompletely understood (Estacio et al., 1998; Fang et al., 2005), and has been attributed to cardiac insufficiency and impaired muscle metabolism (Poirier et al., 2000; Taegtmeyer et al., 2002; Scheuermann-Freestone et al., 2003; Stephens et al., 2007). We consider the effects of functional activation by exercise on the brain and how it contributes to understanding the control of CBF in relation to the limited exercise tolerance experienced by type 2 diabetic patients. Analogies and differences between the cerebral vs. skeletal muscle blood flow responses to exercise are highlighted with emphasis on the dependency of the human brain on the distribution of the available blood flow. A decline in cerebral oxygenation in the later stages of exhaustive exercise may reduce the motor drive to working muscles similar to what is observed during exercise in hypoxia (Rasmussen et al., 2010). Whether a decline in cerebral oxygenation with following reduced neural drive to working muscles plays a role in the development of “central” fatigue during exhaustive exercise is addressed in relation to the altered brain vascular response to exercise in type 2 diabetic patients and their accentuated perceived exertion.

## Autonomic Neural Control of CBF During Exercise

The large increase in systolic blood pressure during exhaustive exercise challenges CBF control mechanisms including cerebrovascular or cerebral autoregulation, the cerebrovascular responsiveness (CVR_CO2_) to carbon dioxide (CO_2_) and oxygen (O_2_) partial pressures, matching of local cerebral blood supply to the metabolic demand (i.e., neurovascular coupling), neurogenic control (Immink et al., 2014; Ritz et al., 2014; Willie et al., 2014; Phillips et al., 2016), and maintenance of cardiac output (Ide et al., 1998, 1999a; Van Lieshout et al., 2001, 2003; Ogoh et al., 2005a; Bronzwaer et al., 2014, 2017). During exercise, CBF increases as quantified by several methods (for review, see Secher et al., 2008; Smith and Ainslie, 2017). Dynamic exercise enhances the transcranial Doppler ultrasound determined middle cerebral artery blood velocity (MCA *V*) and the ^133^Xe clearance determined CBF (Jorgensen et al., 1992) and also the blood flow in the internal carotid and vertebral arteries (Sato et al., 2011). Notably, the increase in CBF during cerebral activation is such that cerebral oxygenation is enhanced as expressed by blood-oxygen-level (BOLD) dependent imaging (Laughlin et al., 2012) and for whole-body exercise, a similar increase in cerebral oxygenation is demonstrated by near-infrared spectroscopy (Ide et al., 1999b). Changes in CBF in response to exercise are restricted to specific areas of the brain and, therefore, blood flow in a single brain artery or vein cannot be considered to fully represent flow to or from the brain as a whole, reflecting that the effects of exercise on brain metabolism are heterogeneous. For example, regulation of internal carotid and vertebral artery flow seems different not only during exercise (Sato et al., 2011) but also during simulated orthostatic stress (Ogoh et al., 2015b). Constancy of diameter of an insonated large cerebral artery is required to link changes in cerebral blood velocity to those in CBF (Coverdale et al., 2014; Verbree et al., 2014, 2017).

Sympathetic activity is proposed to enhance cerebral vascular tone to counteract the increase in cerebral perfusion pressure beyond what is designated as the cerebral autoregulatory range (Purkayastha et al., 2013; Ogoh et al., 2015a), with cerebral perfusion pressure defined as the difference between blood pressure at the level of the circle of Willis and the critical closing pressure, the pressure inside a blood vessel below which it collapses and blood flow ceases. Both sympathetic and cholinergic mechanisms are considered important for restricting the exercise-induced increase in CBF without affecting the cerebral metabolic rate for oxygen (Seifert et al., 2010; Purkayastha et al., 2013; Willie et al., 2014; Ogoh et al., 2015a). Of note, erythropoietin has been applied to improve athletic performance and endurance but it actually reduces cerebrovascular conductance during exercise both under normoxic and hypoxic conditions (Rasmussen et al., 2012). The contribution of autonomic neural control of CBF during exercise remains difficult to detangle (Van Lieshout and Secher, 2008; Mitchell et al., 2009; Willie et al., 2014). The presently available evidence for neurogenic CBF control from rest to exercise is mainly from direct sympathetic ganglion blockade studies. At rest, unilateral trigeminal ganglion stimulation reduces CBF as evaluated by transcranial Doppler ultrasound and by single-photon emission computed tomography (Seifert and Secher, 2011). During exercise, β-adrenergic receptor blockade restricts the increase in cardiac output and in MCA *V* whereas this attenuation is eliminated by stellate ganglion blockade (Ide et al., 1998). Intrinsic cerebrovascular sympathetic activity is indicated by jugular venous “spillover” of norepinephrine from the brain in healthy humans but not in patients with autonomic failure who lack sympathetic vasomotor control (Harms et al., 2000; Mitchell et al., 2009). Apart from these selective investigations numerous studies have manipulated CBF pharmacologically by e.g., angiotensin, α-adrenergic receptor agonists and antagonists, nitric oxide donors, and anesthetic agents (Purkayastha et al., 2013; Willie et al., 2014) but the effects of these interventions on cerebrovascular tone remain controversial (Van Lieshout and Secher, 2008; Willie et al., 2014). For instance, the similarity of reductions in arterial pressure and pulsatile change in MCA *V* before vs. during ganglion blockade while maintaining arterial pressure with phenylephrine was taken to suggest that sympathetic vasoconstriction, mediated through α_2_-adrenergic receptor activation, is not the underlying mechanism for the reduction in CBF during central hypovolemia (Zhang and Levine, 2007). Yet, it should be considered that phenylephrine may lower CBF while increasing mean arterial pressure (Stewart et al., 2013). In diabetic patients, both cerebral autoregulatory capacity (Kim et al., 2008a; Kim, 2014; Vianna et al., 2015) and CVR_CO2_ as the major operative mechanisms maintaining CBF may have become impaired (Dandona et al., 1978; Fulesdi et al., 1997), rendering diabetic patients more susceptible to ischemic episodes (Dandona et al., 1978; Kim et al., 2011).

## Brain Vs. Skeletal Muscle Blood Flow Response to Exercise

A major difference between brain and skeletal muscle is that the brain is active under all living conditions and uses ~15% of cardiac output at rest (Ide et al., 1999a, 2000; Immink et al., 2009; Willie et al., 2014). The effects of exercising in the upright vs. seated position on cardiac preload are exemplified by a higher heart rate in the upright position (Yoshiga and Higuchi, 2002). Equally, the change to the upright posture accompanying the majority of exercise modalities affects both the arterial supply to and the venous drainage from the brain (Van Lieshout et al., 2003; Dawson et al., 2004; Gisolf et al., 2004). When assuming the upright position, global CBF and frontal cortical oxygenation decrease, seemingly at odds with the concept of cerebral autoregulation implicating constancy of CBF for a range of cerebral perfusion pressures. The “constant flow” autoregulation plateau has been constructed from data across different studies rather than quantifying the pressure-flow relationship within individual subjects that, however, is difficult given that the range of blood pressures required for relating flow to pressure remains effectively limited by autonomic cardiovascular reflex activity. Yet, maintaining CBF constant would require an autoregulatory efficacy with an infinite gain, which generally does not apply to biological systems (Van Lieshout et al., 2003; Willie et al., 2014). Obviously, the arterioles rather than large arteries represent the main side of vascular resistance, but also larger arteries contribute to vascular control (Iversen et al., 1995). For the brain, the large extracranial vessels and surface vessels contribute importantly to cerebrovascular resistance, thus being at least passively involved in regulation of CBF (Faraci and Heistad, 1990; Ritz et al., 2014; Willie et al., 2014).

The brain with its small vascular bed being tightly controlled takes up to ~25% of whole-body oxygen consumption at rest (Braz and Fisher, 2016). The vulnerability of the brain is exemplified by the fact that its function deteriorates when cerebral oxygenation is reduced by more than about 10% from the resting level, in contrast to skeletal muscles, that continue their activity despite an O_2_ desaturation below 10% (Quistorff et al., 2008; Secher et al., 2008). Continued exhaustive exercise evokes a competition for the supply of oxygenated blood between the brain and the working muscles. The brain activates the muscles, but from then on, the large increase in muscle blood flow and thus skeletal muscle vascular conductance represents a major competitor for continuous provision of oxygen and substrate upon which the brain relies (Secher et al., 2008). Heavy exercise with large muscle groups requests more blood than the heart can provide and thus requires tight sympathetic vasomotor control to maintain arterial pressure (Calbet et al., 2004). When humans exercise at maximal intensity, up to ~80–90% of total cardiac output is being distributed to skeletal and cardiac muscle (Laughlin et al., 2012). At the same time, an increase in regional CBF has to match the enhanced neuronal metabolism exemplified by an elevated cerebral metabolic rate for oxygen at that stage of exercise (Laughlin et al., 2012). Within the brain, in contrast to skeletal muscles, there is no capillary recruitment and creating and maintaining an elevated O_2_ gradient is a prerequisite given that the efficacy for O_2_ extraction by the brain compared to skeletal muscle is small.

## Cardiac Output Supports CBF During Exercise

The size of cardiac output is important for regulation of CBF beyond arterial pressure both at rest and during exercise (Hellstrøm et al., 1994, 1996; Magnusson et al., 1997; Ide et al., 1998, 1999a,b, 2000; Gruhn et al., 2001; Van Lieshout et al., 2001; Ogoh et al., 2005a; Secher et al., 2008; Braz and Fisher, 2016). In consequence, an incompetence to increase cardiac output sufficiently during exercise may jeopardize cerebral perfusion and thereby the ability of the central nervous system to recruit and adequately drive the motoneurons. The role of cardiac output for distribution of flow is illustrated in patients with moderate heart failure for whom peak skeletal muscle perfusion is maintained, provided that the activated muscle mass is small. Involvement of a larger muscle mass, however, reduces peak leg blood flow, perfusion, and oxygen uptake (Magnusson et al., 1997). Similarly in these patients during one-legged exercise, MCA *V* is maintained but declines with two-legged exercise and exposes a competition between brain and skeletal muscle (Hellstrøm et al., 1996). Thus, the traditional concept that the brain is at the top of the hierarchy of competing physiological needs is challenged when cardiac output no longer matches tissue O_2_ requirements. Under these circumstances, exercise evokes cerebral deoxygenation, metabolic changes, and indices of fatigue similar to those observed during exercise in hypoxia (Secher et al., 2008; Rasmussen et al., 2010). Thus, reduced cerebral oxygenation may play a role for the development of central fatigue as an exercise capacity limiting factor (Rasmussen et al., 2010; Kim et al., 2015).

## Exercise and Brain Vascular Control in Type 2 Diabetes

Physiological aging is associated with a decline in resting cerebral metabolism, global CBF, and gray matter flow but does not in itself implicate affected CBF control (for review, see Braz and Fisher, 2016). Specifically, the normal development of an initial increase in CBF in response to exercise is well maintained in the elderly (Laughlin et al., 2012; Fisher et al., 2013; Braz and Fisher, 2016). During maximal exercise in healthy humans, fatigue is preceded by reductions in systemic and skeletal muscle blood flow, and O_2_ delivery and uptake (Gonzalez-Alonso et al., 2004).

**Figure 1:**
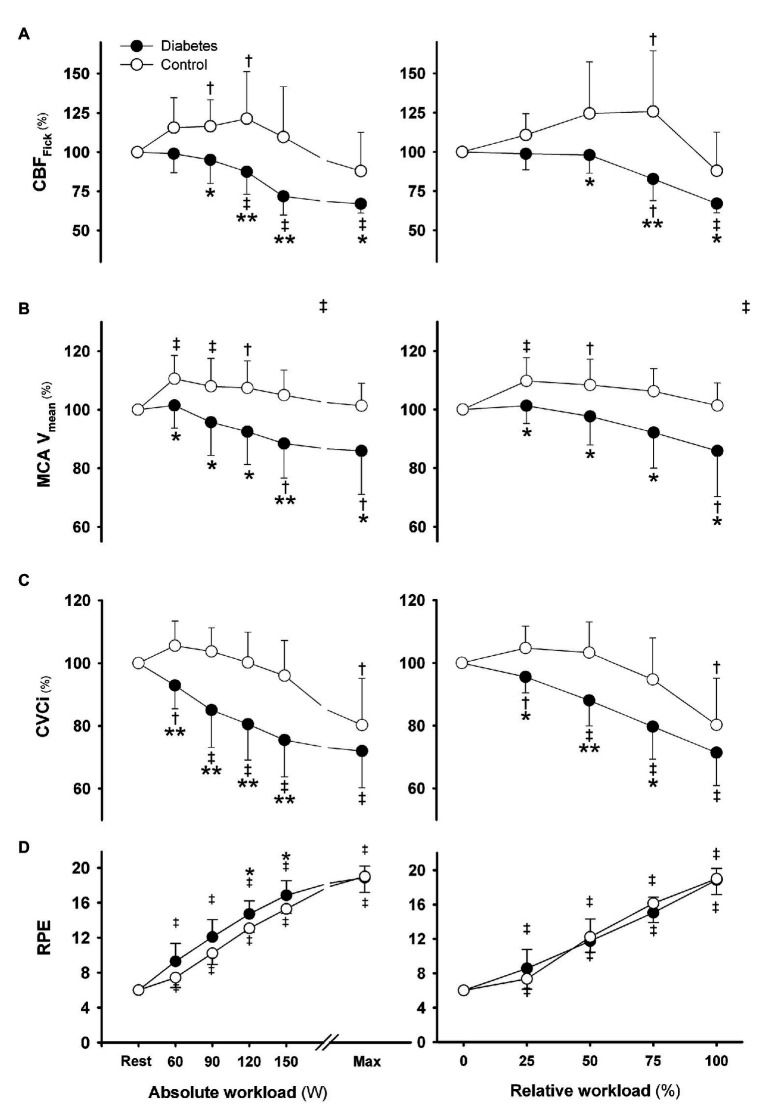
Cerebrovascular response to exercise in eight male type 2 diabetic patients without symptomatic cardio-vascular disease (closed circles) vs. seven age and gender matched healthy subjects (open circles) at the same absolute (left panels) and relative workload (right panels). **(A)** Cerebral blood flow derived from the Fick principle (CBF_Fick_) from inverse arterial-jugular venous oxygen difference, **(B)** middle cerebral artery mean blood flow velocity (MCA V_mean_), **(C)** cerebrovascular conductance index (CVCi), and **(D)** rating of perceived exertion (RPE; Borg scale). The patients demonstrated a decline in cerebral perfusion and oxygenation during incremental exercise associated with attenuated increases in cerebral and systemic vascular conductance compared with healthy controls. Cerebral oxygenation reached its lowest level at exhaustion at a 20% lower workload in type 2 diabetes mellitus (T2DM) patients than healthy controls and patients expressed a higher RPE than healthy controls. ^†^*p* < 0.05 and ^‡^*p* < 0.01 vs. rest; ^*^p < 0.05 and ^**^p < 0.01 vs. control subjects. Values are mean ± SD (modified from Kim et al., 2015).

**Figure 2:**
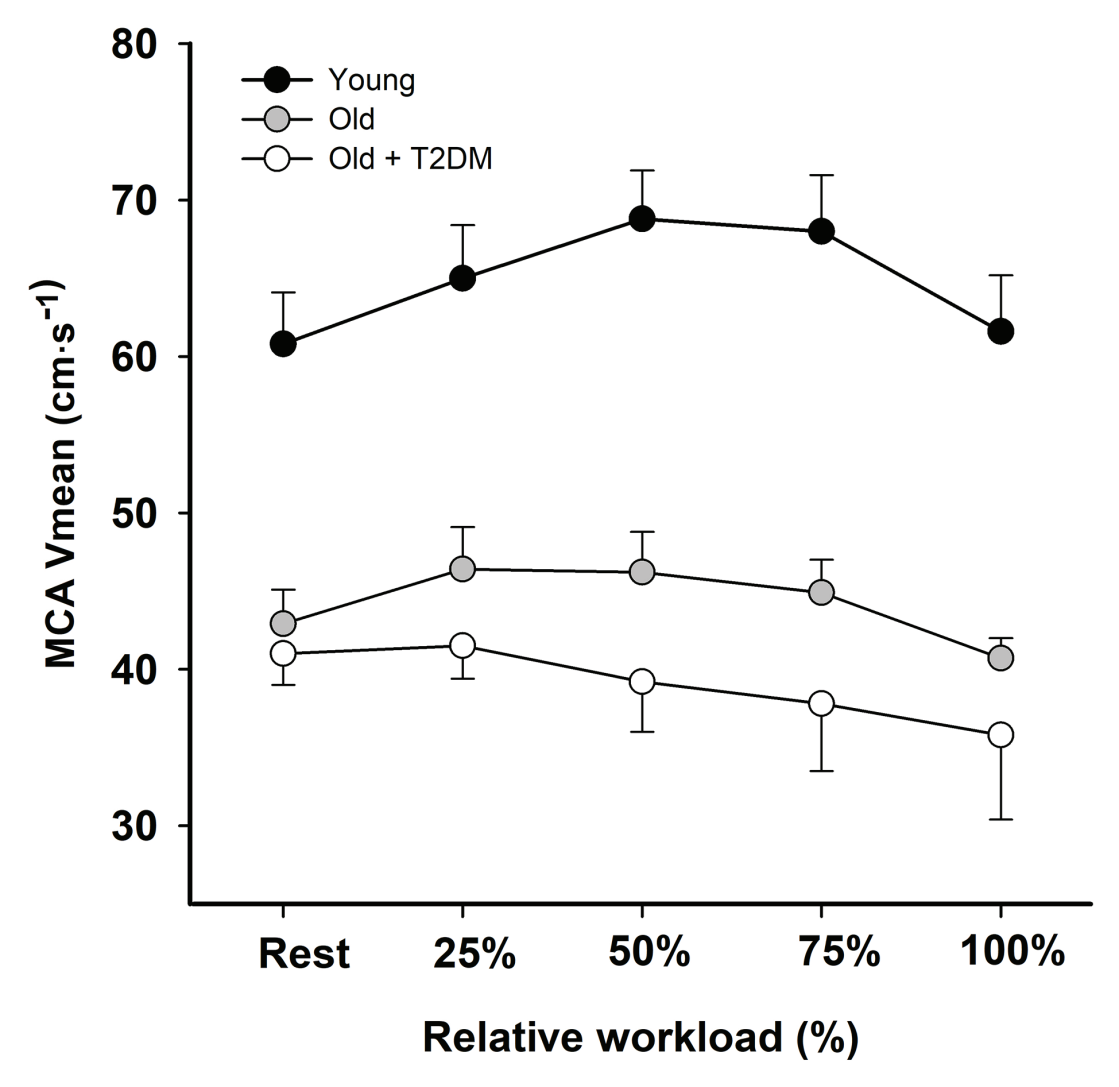
Middle cerebral artery mean blood flow velocity (MCA V_mean_) at rest and during exercise at the same relative workload in young individuals (black circles), and in middle-aged subjects without (gray circles), and with type 2 diabetes (T2DM; open circles). During submaximal and during maximal exercise, cerebral perfusion was reduced in older individuals compared with young individuals, and the more so in the older diabetic patients. Values are mean ± SEM (adapted from Fisher et al., 2013; Kim et al., 2015).

In middle-aged type 2 diabetic patients, the cardiac output reserve and work capacity are low and the increase in CBF that is present in healthy young and elderly does not develop (Figures 1, 2; Kim et al., 2015). Accordingly, these patients demonstrate an early reduction in cerebral oxygenation despite a larger brain O_2_ extraction, and they express enhanced perceived exertion, signifying a fundamental problem in brain vascular control during exercise (Kim et al., 2008a; Vianna et al., 2015). Yet, for these patients, the brain uptake of lactate and glucose is similar to what is found in healthy reference subjects (Kim, 2014; Kim et al., 2015), which points to cerebrovascular rather than brain metabolic derangement. In contrast to the vast amount of studies on the muscle blood flow response to exercise in type 2 diabetic patients, data on the CBF response to exercise in these patients are very sparse. In diabetic patients, progression of microvascular disease interferes with the physiological nocturnal decline in blood pressure, coinciding with a persistently increased arterial pulse pressure and reduced baroreflex sensitivity, contributing to their increased cardiovascular risk (Kim et al., 2019). Treatment of hypertension as a common comorbidity in type 2 diabetes is required to reduce the risk of hypertensive surges during strenuous exercise that challenge the brain vasculature, but intensive blood pressure control may, in contrast to nondiabetic hypertensive patients, reduce their CBF (Kim et al., 2011).

## From Deconditioning to Physical Exercise – a Challenge of Brain Vascular Control

Loss of skeletal muscle mass is a main factor for the increased incidence of type 2 diabetes with aging. Deconditioning as a result of physical inactivity vs. resistance exercise is associated with opposing adaptive responses. Resistance exercise provides better metabolic control (Baldi and Snowling, 2003), mitigates disuse-associated tendon stiffness, maintains or increases skeletal muscle mass, and improves whole body glucose disposal (Fenicchia et al., 2004). Thus, a focus on resistance exercise has been recommended for type 2 diabetic patients, specifically for the subgroup of sarcopenic or severely deconditioned older patients (Sigal et al., 2006). Resistance vs. endurance exercise has different cardiovascular effects. Resistance-type activities produce a considerably larger increase in arterial pressure, because of the mechanical compression of blood vessels together with repeated Valsalva-like maneuvers (MacDougall et al., 1985). Unlike aerobic exercise, resistance training affects central arterial compliance in healthy men (Miyachi et al., 2004).

In healthy young adults, isometric resistance exercise with vs. without concomitant straining produces a greater cerebrovascular challenge (Perry et al., 2020), whereas straining dominates the central and cerebral hemodynamic response to intense static exercise (Pott et al., 2003). Although acute changes in arterial blood pressure during physiological challenges are transmitted to the cerebral circulation, under normal conditions, CBF returns to its baseline value within a few seconds (Panerai et al., 2001; Pott et al., 2003; Immink et al., 2005; Labrecque et al., 2020). Cerebral vasoconstriction constantly plays a protective role during exercise of moderate to heavy intensity, in particular when pulse pressure exceeds the autoregulatory range (Ogoh et al., 2005b). When autoregulatory mechanisms are failing (Immink et al., 2005; Kim et al., 2008a; Frosch et al., 2017; Vranish et al., 2020) or overwhelmed by acute blood pressure surges beyond the autoregulatory range, e.g., grave hypertension, CBF becomes more directly related to its perfusion pressure, resulting in cerebral hyperperfusion manifested by retinal edema and encephalopathy (Immink et al., 2004).

## Perspective

In the European Union, 55 million individuals suffer from type 2 diabetes and 66 million have impaired glucose tolerance, with an estimated ~4% annual increase. Optimizing metabolic control by behavioral modification including regular physical activity, thus slowing down progression of vascular disease is a task for diabetes care. From that point of view, physical activity represents a “medicine” for metabolic disease (Pedersen and Saltin, 2015; Pedersen, 2019). The challenge to optimize metabolic control in individuals with type 2 diabetes may be achieved at least in part by behavioral modification including regular physical activity (Kim et al., 2008b; Pedersen, 2017). Indeed, physical activity by patients with type 2 diabetes markedly improves the impaired insulin action and is considered a cornerstone in the treatment along with diet and medication. Unfortunately, however, type 2 diabetic patients perceive sustained aerobic exercise to be more strenuous than healthy, non-diabetic subjects. This sets a limit to the effectiveness of physical activity as a preventive lifestyle intervention for this patient population (Praet and van Loon, 2008, 2009; Huebschmann et al., 2009, 2015; Nadeau et al., 2009; Regensteiner et al., 2014; Senefeld et al., 2020). Left ventricular diastolic dysfunction may be an early manifestation of diabetic cardiomyopathy. When cardiac function deteriorates, the blood supply to the brain seems no longer safeguarded, pointing to the hitherto underexposed functional connection between heart and brain. Aerobic exercise itself may reveal arterial dysfunction associated with latent and overt cerebrovascular disease (Robertson et al., 2019).

In mice, exercise training increased brain mitochondrial biogenesis (Steiner et al., 2011) and a liver-to-brain axis was identified by which plasma glycosylphosphatidylinositol-specific phospholipase could transfer the benefits of exercise on neurogenesis in the brain from young to old mice (Ansere and Freeman, 2020; Horowitz et al., 2020). Nevertheless, regular physical exercise arguably continues to remain the most consistently effective health-enhancing strategy to attenuate the deterioration in brain structure and function related to aging and type 2 diabetes (Hillman et al., 2008; Mayhan et al., 2011; Espeland et al., 2018; Pedersen, 2019). When applying the concept that failure in regulation at multiple levels is common in diseases like diabetes (Pedersen and Saltin, 2015), a limited ability to increase cardiac output together with reduced systemic and cerebral vasodilatory capacity become primary targets for prevention and treatment, challenging integrative physiologists and clinicians alike.

## Author Contributions

Y-SK contributed to the experimental design, data acquisition, data analysis, and writing the manuscript. BS contributed to data analysis and manuscript revision. PB contributed to manuscript writing. NS contributed to experimental design of studies and writing. JL supervised the study and contributed to the experimental design, data analysis, and writing the manuscript. All authors contributed to the article and approved the submitted version.

### Conflict of Interest

The authors declare that the research was conducted in the absence of any commercial or financial relationships that could be construed as a potential conflict of interest.
